# Preoperative Partial Breast Irradiation in Patients with Low-Risk Breast Cancer: A Systematic Review of Literature

**DOI:** 10.1245/s10434-023-13233-9

**Published:** 2023-03-03

**Authors:** Yasmin A. Civil, Lysanne W. Jonker, Maartje P. M. Groot Koerkamp, Katya M. Duvivier, Ralph de Vries, Arlene L. Oei, Berend J. Slotman, Susanne van der Velde, H. J. G. Desirée van den Bongard

**Affiliations:** 1 Department of Radiation Oncology, Amsterdam UMC Location Vrije Universiteit Amsterdam, Amsterdam, The Netherlands; 2 Cancer Center Amsterdam, Cancer Treatment and Quality of Life, Amsterdam, The Netherlands; 3 Department of Radiology, Amsterdam UMC Location Vrije Universiteit Amsterdam, Amsterdam, The Netherlands; 4 Medical Library, Vrije Universiteit, Amsterdam, The Netherlands; 5 Department of Radiation Oncology, Amsterdam UMC Location University of Amsterdam, Amsterdam, The Netherlands; 6 Laboratory for Experimental Oncology and Radiobiology, Amsterdam UMC Location University of Amsterdam, Amsterdam, The Netherlands; 7 Center for Experimental Molecular Medicine, Amsterdam UMC Location University of Amsterdam, Amsterdam, The Netherlands; 8 Cancer Center Amsterdam, Cancer Biology and Immunology, Amsterdam, The Netherlands; 9 Department of Surgery, Amsterdam UMC location Vrije Universiteit Amsterdam, Amsterdam, The Netherlands

## Abstract

**Background:**

Preoperative instead of standard postoperative partial breast irradiation (PBI) after breast-conserving surgery (BCS) has the advantage of reducing the irradiated breast volume, toxicity, and number of radiotherapy sessions and can allow tumor downstaging. In this review, we assessed tumor response and clinical outcomes after preoperative PBI.

**Patients and Methods:**

We conducted a systematic review of studies on preoperative PBI in patients with low-risk breast cancer using the databases Ovid Medline, Embase.com, Web of Science (Core Collection), and Scopus (PROSPERO registration CRD42022301435). References of eligible manuscripts were checked for other relevant manuscripts. The primary outcome measure was pathologic complete response (pCR).

**Results:**

A total of eight prospective and one retrospective cohort study were identified (*n* = 359). In up to 42% of the patients, pCR was obtained and this increased after a longer interval between radiotherapy and BCS (0.5–8 months). After a maximum median follow-up of 5.0 years, three studies on external beam radiotherapy reported low local recurrence rates (0–3%) and overall survival of 97–100%. Acute toxicity consisted mainly of grade 1 skin toxicity (0–34%) and seroma (0–31%). Late toxicity was predominantly fibrosis grade 1 (46–100%) and grade 2 (10–11%). Cosmetic outcome was good to excellent in 78–100% of the patients.

**Conclusions:**

Preoperative PBI showed a higher pCR rate after a longer interval between radiotherapy and BCS. Mild late toxicity and good oncological and cosmetic outcomes were reported. In the ongoing ABLATIVE-2 trial, BCS is performed at a longer interval of 12 months after preoperative PBI aiming to achieve a higher pCR rate.

**Supplementary Information:**

The online version contains supplementary material available at 10.1245/s10434-023-13233-9.

Female breast cancer is the most commonly diagnosed cancer in the world.^1^ The incidence of early-stage breast cancer is increasing due to the implementation of nationwide mammographic screening programs, improvements in imaging, and aging.^2,3^ Standard treatment for these patients is breast-conserving surgery (BCS) followed by whole breast irradiation (WBI).^4–8^ Since it is known that the majority of recurrences occur in or close to the tumor bed, European and American oncological societies recommended the use of partial breast irradiation (PBI) as an alternative to WBI after BCS in patients classified as low risk.^9–12^ Similar results for 5-years local recurrence are reported for PBI and WBI [1.8%, 95% confidence interval (CI) 0.68–3.2% versus 2.5%, 95% CI 0.92–2.4%].^10^ Radiation-induced toxicity, such as radiation dermatitis, hyperpigmentation, breast edema, fibrosis, pain, and cardiovascular disease, impacts cosmetic outcomes and quality of life of patients after WBI.^13–16^ The advantage of PBI is that it limits the irradiated volume to the tumor bed and decreases toxicity rates due to the irradiation of less breast tissue.^17,18^

However, conflicting results have been published regarding postoperative PBI, as suboptimal cosmetic outcomes and relatively high toxicity rates have also been reported.^19–22^ These outcomes could be attributed to the relatively large dose to surrounding healthy breast tissue due to the uncertainty of postoperative tumor bed definition, as the postoperative irradiated breast volume increases due to surgical artifacts including seroma.^23^ Preoperative irradiation has the advantage of a more precise target definition since the tumor is still in situ, and can result in a reduced irradiated volume, a higher dose per radiotherapy fraction, and thereby fewer radiotherapy fractions.^24,25^ This may reduce the risk of radiotherapy-related toxicity and improve cosmetic outcomes compared with postoperative radiotherapy. In addition, preoperative PBI allows tumor downstaging. This systematic review aims to provide an overview of the existing studies on preoperative PBI in patients with low-risk breast cancer and to assess the clinical outcomes including pathologic complete response (pCR), radiological response, local recurrence, survival, toxicity, and cosmetic outcome.

## Patients and Methods

### Search Strategy

This review is reported according to the Preferred Reporting Items for Systematic Reviews and Meta-Analyses (PRISMA) (https://www.prisma-statement.org/).^26^ A systematic literature search was performed to identify relevant publications in the bibliographic databases Ovid Medline, Embase.com, Web of Science (Core Collection), and Scopus from inception up to 1 December 2022, in collaboration with a medical information specialist. The following terms were used (including synonyms and closely related words) as index terms or free-text words: “Breast neoplasms,” “Radiotherapy,” “Preoperative,” “Partial.” The references of the identified manuscripts were searched for relevant publications. Duplicate manuscripts were excluded. All languages were accepted. The full search strategies for all databases can be found in Appendix A/Supplementary material.

### Eligibility Criteria

All randomized controlled trials, longitudinal observational studies, case–control studies, and retrospective and prospective cohort studies that investigated preoperative PBI delivered by external beam radiotherapy, intraoperative radiotherapy (IORT), or brachytherapy followed by BCS were included in this review. Case reports, case series (fewer than ten patients), editorials, commentaries, and reviews of literature were excluded. If overlap between study populations was identified, the most recent or most complete article was included in this systematic review to prevent duplication bias. When studies had overlap between study populations but reported different outcomes, both studies were included. Studies published in English, Dutch, or French were considered eligible for this review.

### Outcome Measures

The primary outcome was the pCR rate. Secondary outcomes were radiological response, biomarker response, local and regional recurrence rate, overall survival, breast cancer-specific survival, distant metastases-free survival, acute and late toxicity, cosmetic outcomes, quality of life, and (semi)quantitative parameters on magnetic resonance imaging (MRI).

### Study Selection

Study selection was performed blindly by two independent reviewers (Y.C., L.J.). First, the screening of the title and abstract of all manuscripts according to the predefined eligibility criteria was performed. Second, the full texts of the eligible manuscripts were screened. Eligibility disagreements were discussed and resolved with an independent author (D.v.d.B.). The study methodology was registered in the PROSPERO, International Prospective Register of Systematic Review (CRD42020148713, https://www.crd.york.ac.uk/prospero/display_record.php?ID=CRD42022301435).

### Data Extraction

Data extraction was performed by Y.C., and the accuracy of the extracted data was verified by L.J. The following variables were extracted from the eligible manuscripts: year of publication, study design, the number of patients included, duration of follow-up, demographics of patient population, radiotherapy treatment characteristics, time interval between radiotherapy and surgery, type of surgery, acute (< 3 months) and late (> 3 months) toxicity, cosmetic outcome, pathological and radiological response rates, and oncological outcomes.

### Risk of Bias Assessment

The risk of bias in the cohort studies was assessed using the ROBINS-I tool,^27^ a tool for assessing the risk of bias in nonrandomized studies of interventions. The domains of bias assessed with this tool are bias due to confounding, selection of participants, classification of interventions, deviations from intended interventions, missing data, measurement of outcomes, and selection of reported results. Two reviewers assessed the risk of bias independently (Y.C., L.J.). Discrepancies were resolved with an independent author (D.v.d.B.).

## Results

**Fig. 1:**
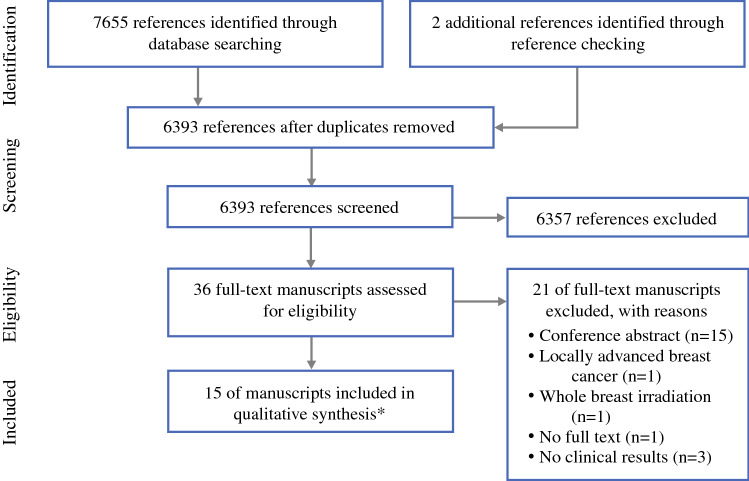
PRISMA Flow chart of the study selection process. *These 15 manuscripts include 6 studies with overlap in study population and are included due to reporting of different outcomes

The literature search generated a total of 7655 references: 1683 in Ovid Medline, 5552 in Embase.com, 178 in Web of Science, and 242 in Scopus. After removing duplicates of references that were selected from more than one database, 6393 references remained and were screened for eligibility. Two manuscripts^28,29^ were found through reference searching. Full texts of 36 manuscripts were screened, and 21 manuscripts were excluded because they were a conference abstract, included patients with locally advanced breast cancer, preoperative WBI was performed, no full text was available, or no clinical results were presented. In total, 15 manuscripts^28–42^ of nine studies were included for qualitative synthesis. The PRISMA flow chart of the search and selection process is shown in Fig. 1.

### Risk of Bias

**Fig. 2:**
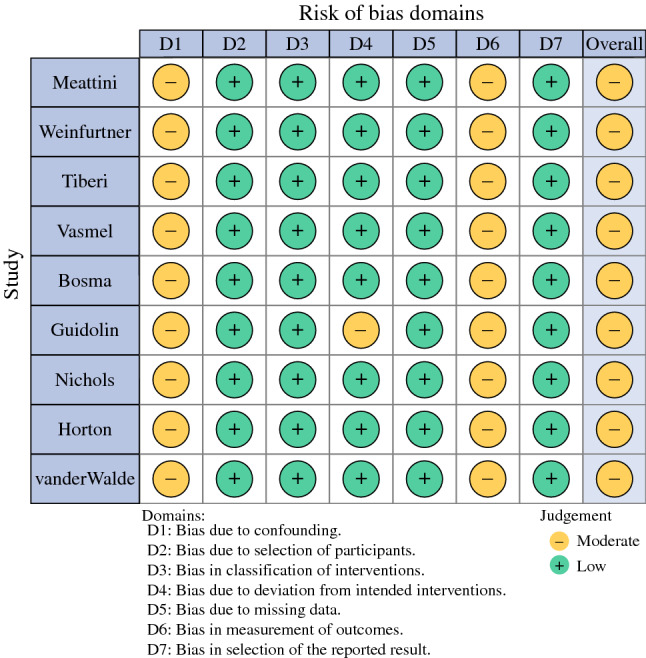
Summary risk of bias assessment with ROBINS-I tool. Low: comparable to a well-performed randomized trial, moderate: sound for a non-randomized study, but not comparable to a rigorous randomized trial, serious: presence of important problems, critical: too problematic to provide any useful evidence on the effects of intervention. Overall risk of bias is equal to the most severe level of bias found in any domain.

Figure 2 shows the risk of bias assessment performed using the ROBINS-I tool.^27^ Bias due to confounding was moderate in all studies, due to the nonrandomized study designs. Blinding of the patients and physicians was not possible in all studies, leading to a moderate risk of bias in measurements of outcome. All other domains were considered at low risk of bias in most studies. In one study, moderate risk of bias due to deviations from intended interventions was scored, as the intervention was not successfully implemented in almost half of the participants.^35^

### Study Characteristics

The 15 manuscripts^28–42^ included in this analysis were published from 2011 to 2022 in North America and Europe. In these manuscripts, eight prospective cohorts and one retrospective cohort were described. Six of these 15 manuscripts^28,30,37,39, 40, 43^ had an overlap of study populations with other included studies but evaluated different outcome measures. Table 1 summarizes the characteristics of the nine studies of which 15 manuscripts were published. In three studies,^31,33,34^ patients were treated using multiple fractions of radiotherapy. In six studies,^29,35, 36,38,41,42^ a single dose of radiotherapy varying between 15 and 21 Gy was administered. The studies included patients with small (< 2–3 cm) unifocal breast tumors with clinically tumor-negative lymph nodes. The time interval between external beam irradiation and breast-conserving surgery ranged between 1 and 32 weeks. An overview of the patient characteristics in all studies is presented in Table 2.

**Table 1:** Characteristics of the included cohort studies on pre-operative partial breast irradiation

Author, year of publication	Technique	Number of patients (*n*)	Dose/fractionation	Inclusion criteria	Outcomes	Time interval to surgery (weeks) (range)
Weinfurtner et al. 2022^a^ ^30,31^	IMRT	19	28.5 Gy/3 fx	≥ 50 years, BC ≤ 2 cm, cN0	Radiologic response, pathologic response	Median 47 days (6–9)
Bosma et al. 2019, 2020^a^ ^33,43^ *PAPBI*	IMRT, VMAT, 3DCRT	133	40 Gy/10 fx in 2 weeks (*n* = 78) 30 Gy/5 fx in 1 week (*n* = 55)	> 60 years, cT1–2 BC, pN0 (sn)	Radiologic response, pathologic response, toxicity, oncological outcomes, cosmetic outcome, quality of life	6
Nichols et al. 2016^34^	3DCRT	27	38.5 Gy/10 fx bid	≥ 18 years, BC < 3 cm, cN0/pN0	Pathologic response, oncological outcomes, toxicity, cosmetic outcome	3
Meattini et al. 2022^42^ *ROCK*	SABR	22	21 Gy/1 fx	≥ 50 years, HR^+^, HER2^−^, unifocal BC ≤ 2.5 cm, cN0	Pathologic response, toxicity, cosmetic outcome	2
Tiberi et al. 2020^41^	IMRT	10	20 Gy/1 fx	≥ 65 years, cT1N0, ER^+^, HER2^−^, grade 1–2	Radiologic response, pathologic response, toxicity	Mean 13
Vasmel et al. 2019^a,b^ ^38,39^ *ABLATIVE*	VMAT	36	20 Gy/1 fx	≥ 50 years, nonlobular, BC ≤ 2 cm (if ≥ 70 years ≤ 3 cm), ER^+^, HER2^−^, cN0	Radiologic response, pathologic response, toxicity, oncological outcomes, cosmetic outcome, quality of life	24 (*n* = 15) 32 (*n* = 21)
Guidolin et al. 2019^a^ ^28,35^ *SIGNAL*	VMAT	27	21 Gy/1 fx	BC < 3 cm, cN0, ER^+^, postmenopausal	Feasibility, toxicity, cosmetic outcome, quality of life	Within 1
Horton et al. 2015^a^ ^36,37^	IMRT	32	15 Gy/1 fx (*n* = 8) 18 Gy/1 fx (*n* = 8) 21 Gy/1 fx (*n* = 16)	≥ 55 years, cT1N0 BC grade 1–2, DCIS ≤ 2 cm, ER/PR^+^, HER2^−^, no lymphovascular invasion	Oncological outcomes, toxicity, cosmetic outcome	Within 10 days
Vanderwalde et al. 2013^c^ ^29^, Kimple et al. 2011^40^ *LCCC* *0218*	IORT	71 (53 only IORT)	15 Gy/1 fx	≥ 48 years, cN0, IDC ≤ 3 cm, visible on ultrasound	Oncological outcomes Toxicity, cosmetic outcome, quality of life	0

*SABR* stereotactic ablative body radiotherapy, *IMRT* intensity-modulated radiation therapy, *VMAT* volumetric modulated arc therapy, *3DCRT* three-dimensional conformal radiation therapy, *IORT* intraoperative radiation therapy, *fx* fractions, *bid* twice a day, *BC* breast cancer, *DCIS* ductal carcinoma in situ, *ER*^*+*^ estrogen receptor positive, *HER2−* Her2-neu receptor negative, *HR*^*+*^ hormonal receptor positive, *IDC* invasive ductal carcinoma.

^a^(Semi)quantitative MRI analysis described in separate article

^b^Neo-adjuvant HT included

^c^Patients treated with only IORT without further local therapy were analyzed

**Table 2:** Patient and tumor characteristics of the included cohort studies on preoperative partial breast irradiation

Author, year of publication	Number of patients (n)	Median age (range)	Median radiologic tumor size (mm) (range)	Grade		Receptor status		pN0	Treatment position	Adjuvant treatment	
Weinfurtner et al. 2022^31^	19	66 (55–77)	12 (6–19)^a^	NR		HR^+^/HER2^−^	100%	79%	NR	NR	
Bosma et al. 2019^33^ *PAPBI*	133	68 (60–87)	13 (5–27)^a^	1 2 3	27% 68% 5%	HR^+^/HER2^−^ HR^−^/HER2^−^ Missing	92% 6% 2%	100%	NR	NR	
Nichols et al. 2016^34^	27	64 (51–85)	11 (0.5–3)^b^	NR		HR^+^/HER2^−^ HR^+^/HER2^+^ HR^−^/HER2^−^	89% 7% 4%	89%	Supine	CT WBI ALND	22% 7% 4%
Meattini et al. 2022^42^ *ROCK*	22	68 (50–86)	14 (8–25)^a^	NR		HR^+^/HER2^−^	100%	86%	Supine	HT HT + CT WBI	82% 14% 9%
Tiberi et al. 2020 ^41^	10	72 (65–84)	8 (0.4–1)^c^	1 2	60% 40%	HR^+^/HER2^−^	100%	90%	Supine	NR	
Vasmel et al. 2019^38^ *ABLATIVE*	36	65 (51–78)	13 (5–20)^a^	1 2 3	67% 25% 6%	HR^+^/HER2^−^	100%	100%	Supine	HT	19%
Guidolin et al. 2019^35^ *SIGNAL*	27	Mean 69 (SD 7)	12 (NR)^c^	1 2 3	44% 52% 4%	HR^+^/HER2^−^ HR^+^/HER2^+^	91% 9%	91%	Prone	NR	
Horton et al. 2015 ^36^	32	66 (55–78)	NR	NR		HR^+^/HER2^−^	100%	78%	Prone (94%) supine (6%)	WBI	9%
Vanderwalde et al. 2013^29^ *LCCC* *0218*	53	63 (48–84)	12 (0.5–2)^c^	1 2 3	38% 38% 24%	HR^+^ HER2^+^ HR^−^/HER2^−^	81% 11% 15%	100%	NR	WBI^d^ Mastectomy	15% 10%

*NR* not reported, *CT* chemotherapy, *WBI* whole breast irradiation, *ALND* axillary lymph node dissection, *HT* hormonal therapy

^a^Tumor size measured on MRI

^b^Tumor size measured on mammogram

^c^Tumor size measured on ultrasound

^d^Patients treated with further local therapy were only included in the analysis of Kimple et al.

### Radiologic and Pathologic Response

**Fig. 3:**
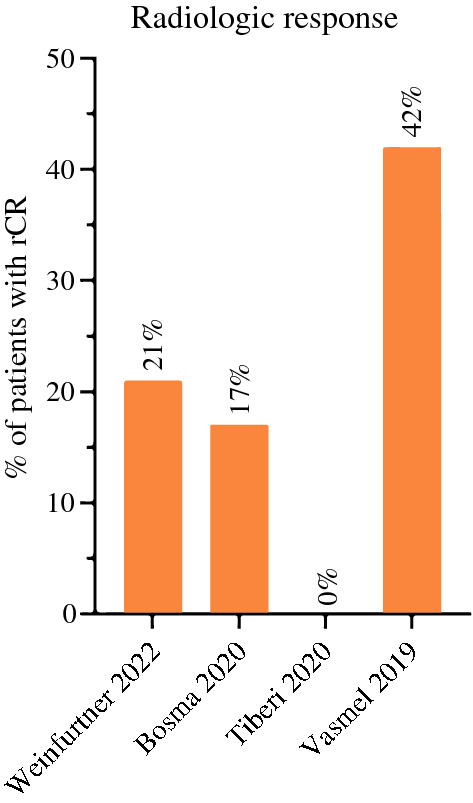
Overview of studies reporting the radiologic complete response rate. rCR: radiologic complete response. The interval between radiotherapy and imaging per study 5–6 weeks (Weinfurtner et al.), 5 weeks (Bosma et al.), 6 weeks (Tiberi et al.) and 24 or 32 weeks (Vasmel et al.)

Four studies^31,38,41,43^ described radiologic complete response (rCR) after preoperative PBI (Fig. 3). Response assessment was performed using MRI in three studies.^31,38,43^ Five to 6 weeks after three fractions of 9.5 Gy, MRI showed a rCR in 21% of the patients.^31^ In the ABLATIVE study^38^ (*n* = 36), MRI was performed 6 (*n* = 15) or 8 (*n* = 21) months after single-dose radiotherapy. The total rCR rate was 42%. In the PAPBI study^43^, radiologic response 5 weeks after PBI (10 × 4 Gy or 5 × 6 Gy) was evaluated on MRI for 48 patients and positron emission tomography (PET)/computed tomography (CT) for 53 patients. The radiologic response on MRI was complete in 17% (*n* = 8) of the patients; seven out of eight patients had a (near) pCR after surgery. On PET/CT a visually complete metabolic response was found in 53% (*n *= 28) of the patients, 7 out of 28 patients had a (near) pCR after surgery. The study of Tiberi et al.^41^ (*n *= 10) used ultrasound to assess response at 6 weeks after a single dose of 20 Gy and showed no rCR.

**Fig. 4:**
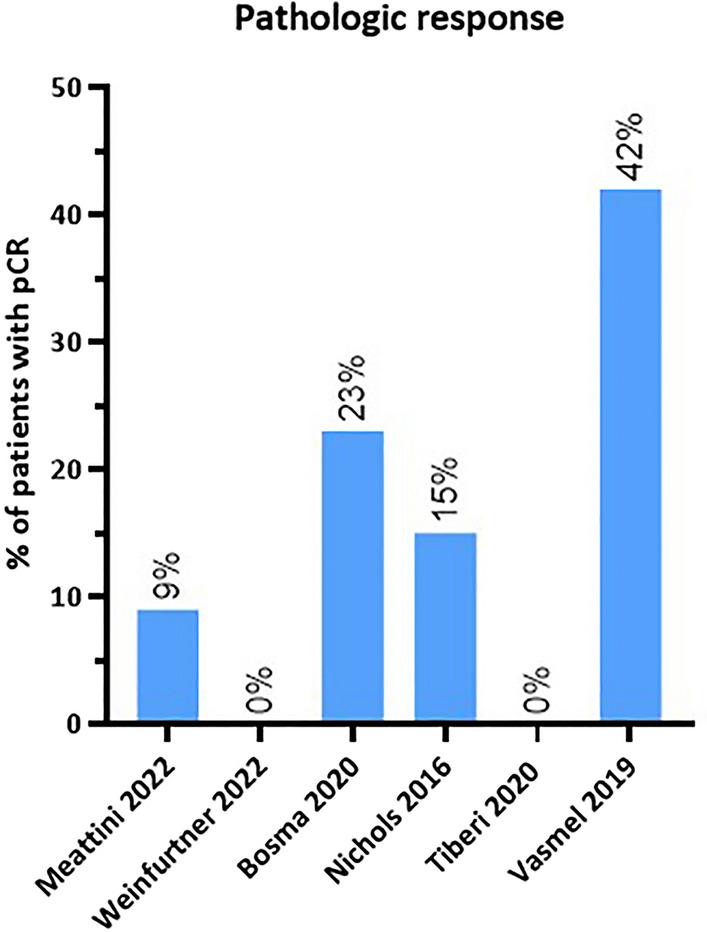
Overview of studies reporting the pathologic complete response rate. pCR: pathologic complete response. The interval between radiotherapy and surgery per study was, 2 weeks (Meattini et al.), 6–8 weeks (Weinfurtner et al.), 6 weeks (Bosma et al.), 3 weeks (Nichols et al.), 11–13 weeks (Tiberi et al.) and 24 or 32 weeks (Vasmel et al.). The pCR rate of Bosma et al. shows a combined rate of complete and near complete response

After surgery, the specimens were assessed on pathologic response in six studies (Fig. 4).^31,34,38,41–43^ The study of Nichols et al.^34^ (*n* = 27) showed a pCR rate of 15% 3 weeks after ten fractions of 3.85 Gy twice daily. The PAPBI trial^43^ found pathologic (near) complete response in 15 out of 66 patients (23%) after 6 weeks. Eight patients had been treated with 10 × 4 Gy and seven patients with 5 × 6 Gy. In the ABLATIVE study,^38^ pCR was achieved in 42% of the patients. Pathologic complete response was observed in 33% and 48% of the patients 6 and 8 months after preoperative PBI, respectively. Near pCR was found in 33% of the patients, partial response in 19%, stable disease in 6%, and none of the patients had an absence of pathologic response. The ROCK trial^42^ reported pCR in two patients (9%) 2 weeks after a single dose of 21 Gy delivered using the Cyberknife robotic system. The remaining two studies^31,41^ showed no pCR after an interval ranging between 6 and 13 weeks, which did not correspond with the radiological response. Tiberi et al.^41^ did show near pCR and partial response in 40% and 40% of the patients, respectively. Two out of 10 patients did not show any tumor response 11–13 weeks after a single dose of 20 Gy. Weinfurtner et al.^31^ reported partial response in 89% of the patients 6–8 weeks after 28.5 Gy in three fractions. Additionally, Nichols et al.^34^ performed tumor response assessment by determination of proliferative activity by immunohistochemical analysis of Ki-67 labeling. The median Ki-67 labeling index was 14% before and 4.2% after radiotherapy (*p* = 0.04).

### Acute Toxicity

Acute toxicity after radiotherapy was evaluated in eight studies (Table 3).^33–36,38,40–42^ Most studies used the common terminology criteria for adverse events (CTCAE) for the classification of toxicity. Two studies^33,42^ used the European Organization for Research and Treatment of Cancer/Radiation Therapy Oncology Group (EORTC/RTOG) criteria. No grade 4 or 5 toxicity was observed. Two studies^33,40^ showed no toxicity in 56% and 94% of the patients. Three studies^34, 35,41^ did not report the rate of grade 1 toxicity. Radiation-induced skin toxicity was the most common acute toxicity. Grade 1 and 2 skin toxicity were reported in respectively 19–34% and 0–10% of the patients.^33,35, 36,38^ Breast seroma was reported in five studies.^33–36,42^ Grade 1 seroma was reported in 0–31% of the patients.^36, 42^ Grade 2 and 3 seroma were reported in 0–11% and 0–15% of the patients, respectively.^34–36, 42^ The PAPBI study^33^ reported seroma in 9% of the patients without any grade specification. The ABLATIVE study^38^ reported overall grade 2 toxicity in 17% of the patients (i.e., breast pain, chest wall pain, arm pain).

**Table 3:** Acute toxicity in the included cohort studies on preoperative partial breast irradiation

Author, year of publication	Number of patients	Skin toxicity (%)	Seroma (%)	Fibrosis (%)	Wound infection (%)	Hematoma (%)	Breast pain (%)	Fatigue (%)
Meattini et al. 2022^b^	22	NR	≥ G1 0	NR	≥ G1 0	≥ G1 0	G1 23 ≥ G2 0	NR
Tiberi et al. 2020^a^	10	G1 UK	NR	NR	NR	NR	NR	NR
Vasmel et al. 2019^a^	36	G1 19	NR	NR	NR	NR	NR	NR
Bosma et al. 2019^b^	133	G1 34 G2 1	9^c^	NR	G2 8 G3 2	G1 1 G3 2	NR	NR
Guidolin et al. 2019^a^	27	NR	≥ G2 0	NR	≥ G2 0	NR	NR	NR
Nichols et al. 2016^a^	27	G1 UK	G2 11 G3 15	G1 UK	G2 7 G3 4	G2 4	G1 UK	G1 UK
Horton et al. 2015^a^	32	G1 28 G2 10	G1 31 G2 0	G1 22 G2 0	G1 0 G2 3	NR	G1 16 G2 6	G1 6 G2 0
Kimple et al. 2011^a^	71	NR	NR	NR	NR	G1 4	G2 1	NR

*UK* unknown, *NR* not reported

^a^Grade (G) according to the common terminology criteria for adverse events (CTCAE)^a^

^b^Grade (G) according to the European Organization for Research and Treatment of Cancer/Radiation Therapy Oncology Group (EORTC/RTOG) criteria

^c^Grade not specified

### Late Toxicity

Six studies^33–36,38,42^ described late toxicity after radiotherapy (Table 4). Grade 1 fibrosis was the most reported late toxicity, in 56–100% of the patients.^33,36,38^ Grade 2 fibrosis was reported in 9% of the patients.^36^ Grade 1 breast discomfort/pain and edema was reported in 13–58% and 31% of the patients, respectively.^33,36,38^ Grade 2 breast pain was reported in 6–13% and grade 3 in 2% of the patients.^33, 36^ Two studies^33,36^ reported grade 1 late skin toxicity in 16–35% of the patients. Grade 2 and grade 3 skin toxicity were reported in 3–7% and 2% of the patients, respectively. Postoperative wound infection in the ABLATIVE trial^38^ was treated with oral antibiotics in 14% of the patients (grade 2), and 3% required surgical intervention (grade 3). The addition of perioperative antibiotics administration to the study protocol prevented new wound infections. Out of 27 patients in the SIGNAL study,^35^ one patient developed grade 2 delayed wound infection. Similarly, Horton et al.^36^ (*n* = 32) reported one patient (3%) with a delayed wound infection. Nichols et al.^34^ observed a fistula in one patient (4%). In the ROCK trial,^42^ grade 1 and grade 2 toxicity was present in respectively 32% and 5% of the patients at 6 months after RT. No grade 2 toxicity was reported at 18 months, and the rate of grade 1 toxicity was 27%. The type of toxicity was not further specified.^42^

**Table 4:** Late toxicity in the included cohort studies on preoperative partial breast irradiation

Author, year of publication	Number of patients	Median follow-up (years)	Fibrosis (%)	Breast pain (%)	Skin toxicity (%)	Wound infection (%)	Seroma (%)	Breast edema (%)	Other toxicity (%)
Vasmel et al. 2019	36	1.8	G1 100%	G1 58%	NR	G2 14% G3 3%	NR	G1 31%	NR
Bosma et al. 2019	133	5.0	G1 UK G2 UK G3 UK	G1 53% G2 13% G3 2%	G1 35% G2 7% G3 2%	NR	NR	NR	Pain rib G1 13%; G2 11%; G3 2% Rib fracture G1 1%; G2 1% Radiation pneumonitis G1 2% Cough G1 3% Teleangiectasia 2%
Guidolin et al. 2019	27	1.0^c^	NR	NR	NR	G2 4%	NR	NR	NR
Nichols et al. 2016	27	3.6	G1 UK	G1 UK	NR	NR	NR	G1 UK	Fistula 4%
Horton et al. 2015	32	1.9	G1 56% G2 9% G3 3%	G1 13% G2 6%	G1 16% G2 3%	G2 3%	G1 6% G2 3%	NR	Breast atrophy G1 6%; G2 6%; G3 3% Fatigue G1 3% Lymphedema G2 3% Pruritus G1 3% Skin hyperpigmentation G1 19%; G2 3% Telangiectasia G1 6%

*UK* unknown, *NR* not reported

^a^Grade (G) grade according to the common terminology criteria for adverse events (CTCAE)

^b^Grade (G) according to the European Organization for Research and Treatment of Cancer/Radiation Therapy Oncology Group (EORTC/RTOG) criteria

^c^One-year follow-up results of all patients are presented (excluding three patients diagnosed with another primary tumor or recurrence)

### Cosmetic Outcome

**Fig. 5:**
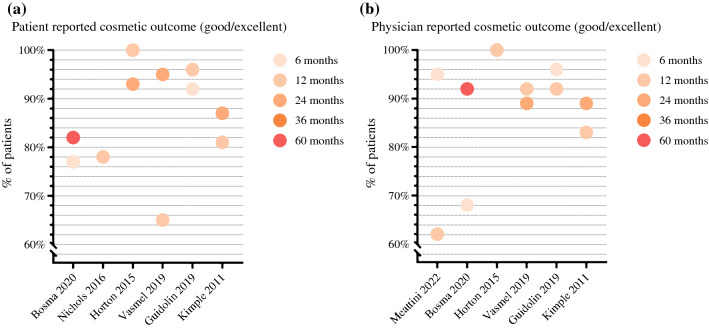
**a** Patient reported cosmetic outcomes. X-axis: studies reporting patient reported cosmetic outcome. Y-axis: the percentage of patients who rated their cosmetic outcome as good or excellent. The legend on the right shows the follow-up duration. *The study by Vasmel et al. reported the percentage of patients who were satisfied or very satisfied with the cosmetic outcome. **b** Physician reported cosmetic outcome. X-axis: studies reporting physician reported cosmetic outcome. Yaxis: the percentage of patients whose cosmetic outcomes were scored as good or excellent by the physician. The legend on the right shows the follow-up duration

Cosmetic outcome was reported in seven studies (Fig. 5).^33–36,38,40,42^ All studies showed that 78–100% of the patients rated the cosmetic result as excellent or good after breast cancer treatment (Fig. 5A). In the ABLATIVE study^38^ cosmetic outcome improved during longer follow-up. Twelve months after treatment, 65% of the patients were satisfied or very satisfied with the cosmetic results, and at 24 months this increased to 95% of the patients. Physicians rated cosmetic outcomes as excellent or good in 62–100% of the patients (Fig. 5B).^33,35,36,38,40,42^ The PAPBI study^33^ also showed an improvement in excellent/good cosmetic outcomes reported by the physician from 68% at 6 months to 92% after 5 years. The ROCK trial^42^ reported a deterioration of cosmetic outcome from 95% scored as good/excellent at 6 months to 62% at 12 months. Physicians rated the cosmetic outcome at 12 months as fair and poor in 24% and 14% of the patients, respectively. In the LCCC 0218 study,^40^ analysis of the 2-years cosmetic results included patients treated with postoperative WBI (*n* = 22) and mastectomy (*n* = 7). The analysis of the 1-year cosmetic results included patients treated with IORT only. Three studies^33,38,42^ evaluated cosmetic outcomes after treatment using the BCCT.core software in which digital photographs of the breast are objectively evaluated. Likewise, the software scored cosmetic outcomes as excellent or good in 82–100% of the patients.

### Oncological Outcomes

Six studies^29,33,34,36,38,42^ reported oncological outcomes after preoperative PBI (Table 5). Horton et al.^36^ showed no breast cancer events in 32 patients during a median follow-up of 1.9 years after single-dose PBI. Similarly, the ROCK trial^42^, the ABLATIVE trial^38^, and Nichols et al.^34^ reported no local recurrences during a median follow-up ranging between 1.5 and 3.6 years. However, one patient from the ABLATIVE trial,^38^ who stopped endocrine therapy prematurely, was diagnosed after 21 months with regional recurrence and distant metastasis in the ipsilateral axillary lymph nodes, vertebrae, and pelvis. The 2-years disease-free survival (DFS) was 97%. The PAPBI trial^33^ reported an ipsilateral breast recurrence rate of 3%. Locoregional recurrence and distant metastasis rates were respectively 2% and 1%. Overall survival rates of the external beam radiotherapy studies ranged between 97 and 100%.^33,38^ The single study on preoperative IORT^29^ showed a higher ipsilateral breast recurrence rate of 15% after a median follow-up of 5.8 years. Three out of 53 patients in this study died of causes not related to breast cancer. Breast cancer-specific and overall survival were 100% and 94.3%, respectively.

**Table 5:** Oncological outcomes per study

Author, year of inclusion	Technique	Number of patients	Median follow-up (years)	Ipsilateral breast recurrence (%)	Locoregional recurrence (%)	Distant metastasis (%)	Overall survival (%)
Meattini et al. 2022	SABR	22	1.5	0	0	0	NR
Vasmel et al. 2019	VMAT	36	1.8	0	3	3	100
Bosma et al. 2019	IMRT, VMAT, 3DCRT	133	5.0	3	2	1	97
Nichols et al. 2016	3DCRT	27	3.6	0	NR	NR	NR
Horton et al. 2015	IMRT	32	1.9	0	0	0	NR
Vanderwalde et al. 2013	IORT	53	5.8	15	2	2	94

*VMAT* volumetric modulated arc therapy, *IMRT* intensity-modulated radiation therapy, *3DCRT* three-dimensional conformal radiation therapy, *IORT* intraoperative radiation therapy, *NR* not reported

### Quality of Life

In total, four studies^33,35,38,40^ reported on the quality of life (QoL) of patients after breast cancer treatment. The ABLATIVE study^38^ used the EORTC-QLQ-C30/-BR23 questionnaires and the Hospital Anxiety and Depression Scale (HADS) to assess QoL. This assessment showed no changes in patient-reported breast symptoms, anxiety, and depression scores before and after breast cancer treatment. The PAPBI study^33^ reported QoL using the EORTC QLQ-C30 and -BR23 questionnaires at baseline and 2 and 4 years after treatment. Mean scores of QoL were similar at all measurement moments. In the SIGNAL study,^35^ a significantly improved QoL score was observed 3 weeks and 1 year postoperatively compared with the baseline QoL score. After preoperative IORT, 88% of the patients were totally satisfied with the treatment received and 5% stated they would not undergo the same treatment again.^40^

### (Semi)quantitative Response Monitoring on MRI

Four studies^28,30,37,39^ evaluated (semi)quantitative parameters to assess treatment response after preoperative PBI using dynamic contrast-enhanced (DCE) and diffusion-weighted imaging (DWI) MRI. In the ABLATIVE study,^39^ the time to enhancement (TTE) increased in radiologic complete responders, the 1-min relative enhancement (RE_1min_) and the percentage of enhancing voxels (%EV) decreased 6 months after preoperative PBI (1 × 20 Gy) compared with baseline. For pathologic complete responders, TTE and the apparent diffusion coefficient (ADC) values increased and %EV decreased at 6 months compared with baseline. No association between the MRI parameters and radiologic and pathologic responses could be proven in this study.

In the SIGNAL trial,^28^ magnetic resonance (MR) images were acquired from a total of 17 patients, 5–7 days after a single fraction of 21 Gy (group 1, *n* = 5), 16–19 days after a single fraction of 21 Gy (group 2, *n* = 6), and 16–18 days after three fractions of 10 Gy every other day (group 3, *n* = 6). Signal-enhancement volumes representing changes in the surrounding breast tissue increased in all patients in group 1 and four out of six patients in group 2. In group 3 a decrease was observed. The mean *K*^trans^ significantly increased in group 1 by 76%, while groups 2 and 3 showed a significant decrease of 15% and 34%, respectively. Mean values of the volume of the extracellular-extravascular space (*v*_e_) in group 1 did not change, and groups 2 and 3 showed an increase of 24% (*p* = 0.043) and 23% (*p* = 0.08).

Wang et al.^37^ found that the initial area under the concentration curve (iAUC) of the contrast agent and *v*_e_ significantly increased in PTV and CTV in 15 patients at 10 days after radiotherapy (1 × 15 Gy/18 Gy/21 Gy). The relative change in regional averaged ADC in GTV and *K*^trans^ in PTV showed statistically significant linear relationships with increasing radiotherapy dose.

Weinfurtner et al.^30^ evaluated the quantitative changes in intratumoral habitats on MR images 5–6 weeks after preoperative irradiation (28.5 Gy/three fractions). Eight tissue types were defined on the basis of the degree of maximum contrast enhancement on MRI and by one of the four DCE-MRI phases in which maximum enhancement was achieved. Quantitative whole breast and tumor percent habitat makeup (%HM) analysis was performed by summing up the number of voxels in each habitat and dividing by the total voxels in the segmented volume (whole breast or tumor). The combined %HM for H1–3 (high enhancing, maximum achieved at dynamic sequence 1–3) decreased by 17% after radiotherapy. This parameter also distinguished patients with a partial pathologic response (%TC ≤ 70%) from patients with no response with an accuracy of 94%, 93% sensitivity, 100% specificity, 100% PPV, and 67% NPV.

## Discussion

This is the first systematic review, to our knowledge, that investigated preoperative PBI alone followed by BCS in patients with low-risk breast cancer. A total of nine studies with 359 low-risk patients were analyzed to evaluate the response and clinical outcomes after preoperative PBI. The results showed an increased rCR and pCR rate up to 42% and 48%, respectively, when the interval between preoperative PBI and BCS was prolonged until 8 months. Radiologic response was not predictive for pCR in all patients treated with preoperative PBI. Recurrence rates were low in almost all studies, except for preoperative IORT. Disease-free and overall survival was high in all studies. Cosmetic outcome was scored as good or excellent in the majority of patients.

A systematic review^44^ on the use of preoperative radiotherapy in locally advanced breast cancer reported three studies^45–47^ on preoperative radiotherapy alone with pCR rates ranging between 10 and 19%. The interval between radiotherapy and surgery in these studies was 4 weeks. In low-risk breast cancer, the pCR rate was higher (up to 48%) as observed in the ABLATIVE trial^38^ with a longer interval of 8 months between radiotherapy and BCS. Although 17% of the patients received neoadjuvant endocrine therapy in the ABLATIVE trial, pCR rates were higher (48%) in the group not treated compared with the group who did receive preoperative endocrine treatment (33%). Two studies^31,41^ found no pCR after preoperative PBI, due to a small study population and a shorter interval between RT and BCS (6–13 weeks).

Tumor response assessment during the interval between radiotherapy and BCS was performed using different imaging modalities. In patients with breast cancer treated with preoperative chemotherapy, MRI is the most accurate imaging modality to assess residual disease and is used as a standard response monitoring tool.^48–50^ In patients treated with preoperative PBI, MRI has a PPV to predict pCR of 67–88% and NPV of 76–85%.^38, 43^ In the study of Tiberi et al.,^41^ ultrasound was performed 6 weeks after radiotherapy and no rCR was found in all ten patients. This may be explained by the fact that ultrasound is less specific for predicting pCR than MRI (57% versus 33%).^51^ On ultrasound, it is difficult to distinguish breast fibrosis from tumor.

Efforts have been made to predict breast tumor response after preoperative chemotherapy by exploration of DCE-MRI and DWI-MRI.^52–56^ DCE-MRI provides extraction of parameters to assess microvascular function such as the rate that reflects the influx of contrast agent into the extracellular-extravascular space (*K*^trans^), which is a measure of capillary permeability, and the fractional volume of the extracellular extravascular space (*v*_e_). The results of our systematic review suggest that MRI-(semi)quantitative parameters could also be used as biomarkers to evaluate tumor response after irradiation. Results from the SIGNAL study^28^ showed a larger *K*^trans^ decrease 3 weeks after 30 Gy in three fractions compared with a single dose of 21 Gy (35% versus 15%) suggesting a stronger radiation response. However, only a single dose of 21 Gy in the SIGNAL study increased the enhanced volume for the surrounding tissue. MRI-based radiomics are a promising non-invasive approach to predict pCR after preoperative therapy and need further exploration in more patients.

Gene expression profiling in patients with breast cancer treated with chemotherapy is widely described in literature and is used to predict tumor response.^57^ Two studies^32,36^ described gene expression changes after preoperative PBI. Analysis of formalin-fixed paraffin-embedded material showed increased gene expression enriched for modulators of the inflammatory and immune response 10 days after preoperative radiotherapy.^36^ In the PAPBI study,^32^ surgery was performed 6 weeks after radiotherapy, and gene expression profiling was performed on fresh frozen tissue. This analysis showed upregulation of the expression of genes involved in pathways of cell death or DNA repair, inflammatory response, and epithelial–mesenchymal transformation. Also, downregulation in the expression of genes involved in the cell cycle was observed. It is suggested that expression patterns of multiple genes could be useful in predicting tumor response after preoperative therapy, as single-gene biomarkers have not been found in previous studies.

Other biomarkers have been investigated to predict pCR after preoperative therapy in patients with breast cancer.^58^ The use of Ki-67 to predict pCR is widely described in literature.^58–60^ A recent meta-analysis^60^ showed a statistically significant difference in pCR rates between high and low Ki-67 expression [odds ratio (OR) 2.94; 95% CI 2.20–3.93]. However, no consensus has been achieved yet on standard values for classifying Ki-67 as high or low. In addition, the Ki-67 proliferation index is significantly associated with the presence of high stromal tumor-infiltrating lymphocytes (TILs), which is an indicator of an antitumor immune response that determines the success of preoperative systemic treatment.^61,62^ In the ABLATIVE study,^63^ TILs were evaluated in tumor tissue before and after irradiation. A significant median decrease was observed in the number of TILs after irradiation compared with the amount before irradiation in 22 patients (CD3^+^ 69%,* p* = 0.002; CD4^+^ 27%, *p* = 0.003; CD8^+^ 74%, *p* = 0.004).^63^ The decreased amount of TILs could be explained by the low number of vital tumor cells in low-risk patients with small breast tumors. No significant difference in pre-irradiation TILs was found between responders and nonresponders, although patient numbers were small. Remarkably, TILs were still observed 6 or 8 months after preoperative treatment.

Oncological outcomes after preoperative PBI using external beam radiotherapy were found to be excellent in this systematic review. Studies reported low recurrence rates (0–3%) and high overall survival (97–100%), which is consistent with the literature on postoperative PBI in patients with low-risk breast cancer.^10,17,18,21,64^ In patients treated with postoperative PBI, the 5-years local recurrence rate was 0.5–4.7% and overall survival ranged between 96.3 and 98.1%. A registry study^65^ on 250,195 women with early-stage breast cancer showed a lower hazard ratio (HR) for second primary cancer among estrogen receptor-positive (ER^+^) patients after preoperative radiotherapy compared with postoperative radiotherapy (BCS: HR 0.64, *p* < 0.0001). It could be hypothesized that this might be caused by the abscopal effect, which means that the radiation of a tumor can activate an antitumor immune response.^66^ In addition, ER^+^ patients with ductal carcinoma in situ had significantly higher incidences of second primary tumors than patients with stage T1 tumors (HR 1.19, *p* < 0.0001).^65^ Progesterone receptor status did not influence the rate of a secondary primary breast cancer.

Almost all studies in the current systematic review used external beam radiotherapy with single or multiple fractions, except for the LCCC 0218 study,^29^ which used preoperative IORT. This treatment led to high rates (15%) of local recurrence after 6 years. In IORT, radiotherapy may not reach far enough into the breast tissue to treat the microscopic spread of tumor cells. Additionally, the use of preoperative ultrasound to determine the depth of tissue irradiation could have led to an underestimation of the margins.^29^ An important prognostic factor in the LCCC 0218 study^29^ is that almost half of the patients were retrospectively classified as Cautionary according to the 2009 ASTRO Consensus Statement on Accelerated Partial Breast Irradiation (APBI).^11^

Another important finding of this study is that acute and late toxicity after preoperative PBI was predominantly mild or moderate. Only a minority of patients (2–3%) with severe acute adverse events, such as wound infection and hematoma, required surgical treatment or intravenous treatment.^33,34,38^ The variation in the number of patients who experienced toxicity across studies may be caused by the use of varying toxicity grading methods, interobserver variability in toxicity scoring, different radiotherapy dose fractionation schedules, and a wide-ranging follow-up duration between 1 and 6 years. The most common definition for acute toxicity was toxicity within 3 months of treatment.^33,36,38^ However, the SIGNAL trial^35^ evaluated acute toxicity at 3 weeks, and two other studies^34,40^ did not specify acute toxicity definition. The ROCK trial^42^ defined acute skin toxicity as an adverse event within 6 months from radiotherapy. This inconsistency and these heterogenic data make comparison with previous data on toxicity after postoperative PBI difficult.^10^

Late treatment-associated toxicity, especially the presence of subcutaneous fibrosis, is correlated with cosmetic outcome.^19,67^ Cosmetic outcome after preoperative PBI was scored by physicians and patients as excellent or good in 62–100% of the cases and improved significantly with longer follow-up after surgery. This could be attributed to the reduction of induration after BCS over time. However, the ROCK trial^42^ showed a deterioration of the cosmetic outcome reported by the physician at 6 and 12 months, which could be partly attributed to previous contralateral breast surgery for benign disease. Studies on postoperative PBI versus WBI report conflicting cosmetic results. Two trials in which patients were treated with postoperative PBI in either 5 or 15 fractions reported improved cosmetic outcomes compared to WBI.^17,18^ In the RAPID and IRMA trial, patients were treated with a twice-daily regimen and had a deterioration of the cosmetic results compared with postoperative WBI.^21, 22^ Sufficient time between radiotherapy fractions is mandatory for normal tissue repair; consequently, a short interval leads to poor cosmetic outcomes.^68,69^ Data of the Danish Breast Cancer Group showed that the frequency of breast induration increases with increasing irradiated breast volumes.^70^ In addition, the proportion of the breast volume receiving 50% or 100% of the prescribed dose is shown to be correlated with cosmetic outcome.^71^ Preoperative PBI is an excellent alternative, and allows irradiation of less healthy breast tissue and tumor downstaging, which could reduce the surgical excision volumes or even omit surgery,^25^ both leading to improved toxicity and improved cosmetic outcome.^71,72^

There are several limitations to the present systematic review. All included studies are observational cohort studies, and RCTs are lacking to date. Also, there is heterogeneity across these studies in radiotherapy regimens, the timing of surgery, administration of systemic therapy, and patient inclusion criteria. Follow-up duration was shorter than 5 years in the majority of studies and varied widely, causing difficulty in comparing toxicity and cosmetic outcome across studies. Short follow-up duration does not permit adequate assessment of late toxicity, cosmetic results, and oncological outcomes, especially since recurrences after (ER^+^) breast cancer can occur up to 32 years after treatment.^73^ Trials with longer follow-up and larger study populations are currently recruiting (Table 6). In the ongoing ABLATIVE-2 trial (NCT05350722), in which patients with low-risk breast cancer are treated with single-dose preoperative PBI, the rate of pCR is assessed using MRI and response markers in blood and tumor tissue. In the ongoing SIGNAL-2 trial (NCT02212860), patients are treated with either a single fraction of 21 Gy or three fractions of 10 Gy and BCS after 5–6 weeks. This study focuses on the pathologic assessment of the impact of radiation on the tumor and immune markers. The study of Tiberi et al.^41^ was an initial analysis of the SPORT-DS trial (NCT03917498), which still has to be completed. If pCR after preoperative PBI can be accurately predicted by combining imaging and biological features, this could allow omission of surgery in future patients. However, a sentinel node procedure is still mandatory to rule out lymph node involvement according to the current clinical practice, since patients with a tumor positive sentinel node are not eligible for PBI, and additional axillary and/or systemic treatment is indicated. If future outcomes of ongoing trials on omission of sentinel lymph node biopsy (NCT02167490, NCT02271828) will show oncological safe results in patients eligible for partial breast irradiation, it could facilitate the implementation of preoperative PBI.

**Table 6:** Summary of the ongoing clinical trials regarding preoperative partial breast irradiation

Trial ID, status	Title	Treatment	Primary endpoints	Secondary endpoints	Estimated primary completion date
NCT05350722, recruiting	Single-dose preoperative partial breast irradiation in low-risk breast cancer patients (ABLATIVE-2)	Preoperative single-dose radiotherapy (20 Gy) and BCS after 12 months	Pathologic complete response	Radiologic complete response, treatment-related adverse events, quality of life, cosmetic outcome, oncological outcomes, immune response, and biomarkers	March 2025
NCT03917498, active/not recruiting	Single pre-operative radiation therapy - with delayed surgery for low risk breast cancer (SPORT-DS)	Preoperative single-dose radiotherapy and BCS after 3 months^a^	Pathologic complete response	Radiation toxicity	28 February 2020 (actual)
NCT02212860, active/not recruiting	Stereotactic image-guided neoadjuvant ablative radiation then lumpectomy (SIGNAL 2)	Preoperative PBI (21 Gy or 3× 10 Gy) and BCS after 14–20 days	Immune priming, angiogenesis, proliferation/hypoxia/apoptosis/invasion markers, toxicity	Cosmetic outcome, survival	April 2021 (actual)
NCT04679454, recruiting	Single fraction preoperative radiotherapy for early stage breast cancer (CRYSTAL)	Preoperative single dose radiotherapy (18 Gy, 21 Gy, 24 Gy) and BCS after 4–8 weeks	Dose escalation, pathologic complete response	Chronic toxicity, cosmetic outcome, postoperative complications, oncological outcomes	March 2026
NCT03909282, recruiting	Phase 2 surgical excision vs neoadjuvant radiotherapy+delayed surgical excision of ductal carcinoma (NORDIS)	Preoperative PBI (5× 6 Gy) and BCS after 3 months versus upfront surgery	Rate of DCIS pathologic complete response	Wound complication, correlation of imaging characteristics and pathologic findings, rate of invasive carcinoma	September 2024
NCT04040569, recruiting	A phase I dose escalation study of single fraction pre-operative stereotactic partial breast irradiation (S-PBI) for early stage breast cancer	Preoperative single dose radiotherapy (30 Gy, 34 Gy, 38 Gy) and BCS^b^	Dose escalation, cosmetic outcome	–	September 2024
NCT02482376, active/not recruiting	Preoperative single-fraction radiotherapy in early stage breast cancer	Preoperative single-dose radiotherapy (21 Gy) and BCS^b^	Physician-reported cosmetic outcome	Ki-67, patient-reported cosmetic outcome, gene expression, local control, circulating cell free DNA	March 2025

*BCS* breast-conserving surgery, *PBI* partial breast irradiation

^a^Dose not reported

^b^Timing of surgery not specified

Patients with no pCR could still benefit from preoperative irradiation since the number of radiotherapy fractions is reduced to one fraction instead of the standard multifractionated radiotherapy schedule (i.e., 5–25 postoperative fractions).^74–76^ Reducing the number of fractions will improve healthcare logistics, decrease healthcare costs, and reduce the treatment burden for patients.^77^ Single- or three-fraction postoperative PBI with brachytherapy and pencil-beam scanning proton PBI has previously proven to be feasible with low toxicity rates.^78–80^ However, these techniques are not widely available and used only in study context. The most common type of radiation treatment is photon beam radiation therapy. Single-dose external photon beam postoperative PBI has been studied in patients with low-risk ductal carcinoma in situ (DCIS) and breast cancer at the University of Washington and was feasible and safe after a median follow-up of 25 months.^81^ Longer follow-up is required, although preoperative PBI has more advantages compared with postoperative PBI. Preoperative PBI allows a more precise definition of the irradiated target volume with less interobserver variability among radiation oncologists, reduced irradiated target volumes, less setup uncertainty on the linear accelerator, and tumor downstaging.^25,82^ Consequently, preoperative PBI could result in lower toxicity and better quality of life.

Several randomized trials have investigated the omission of postoperative WBI after BCS in patients treated with postoperative endocrine therapy.^83–86^ Omission of postoperative WBI and treatment with solely endocrine therapy has reduced the number of local recurrences to a lesser extent compared with WBI. In addition, endocrine therapy can decrease the quality of life of patients with breast cancer, and adherence is around 66% after 5 years.^87,88^ Consequently, preoperative radiotherapy instead of postoperative radiotherapy could lead to de-escalation of breast cancer treatment without increasing treatment-related toxicity. Results of the ongoing Tailored treatment in Older Patients (TOP-1) study (BOOG study number 2016-01) will provide more information on the absolute locoregional recurrence risk in elderly patients in whom postoperative PBI is omitted without adjuvant endocrine treatment.

## Conclusion

This systematic review provided an overview of the existing literature on preoperative PBI in patients with low-risk breast cancer. A longer interval between radiotherapy and surgery increases the rCR and pCR rate after preoperative PBI. Preoperative PBI leads to acceptable toxicity and good to excellent cosmetic outcomes. All studies on preoperative external beam PBI reported low recurrence and high overall survival. In future patients with accurately predicted pCR, preoperative PBI could lead to the omission of BCS and more personalized patient care.

## Supplementary Information

Below is the link to the electronic supplementary material.

Supplementary file1 (DOCX 15 KB)
