# Supplementary material for: Preoperative Partial Breast Irradiation in Patients with Low-Risk Breast Cancer: A Systematic Review of Literature
Source: Ann Surg Oncol. 2023 Mar 3;30(6):3263–79. doi: 10.1245/s10434-023-13233-9 (PMC10175515; doi:10.1245/s10434-023-13233-9)
Supplement: Supplementary file 1 — Supplementary file1 (DOCX 15 KB) [file 10434_2023_13233_MOESM1_ESM.docx]

**Supplementary File**

1. **Full search strategy**

**Ovid Medline Session Results (01 Dec 2022)**

| Search | Query | Items found |
| --- | --- | --- |
| #4 | **1 AND 2 AND 3** | 1,683 |
| #3 | **exp Preoperative Period/ OR exp Neoadjuvant Therapy/ OR preoperative.ab,ti,kf. OR pre-operative.ab,ti,kf. OR before-surg*.ab,ti,kf. OR neoadjuvant*.ab,ti,kf. OR neo-adjuvant*.ab,ti,kf.** | 404,628 |
| #2 | **exp Radiotherapy/ OR radiotherapy.fs OR ((target* OR partial* OR segmental) ADJ3 (radiotherap* OR radiati* OR irradiati* OR x-ray-therapy OR x-ray-therapies)).ab,ti,kf.** | 312,074 |
| #1 | **exp Breast Neoplasms/ OR ((exp Breast/ OR breast*.ab,ti,kf. OR mamma*.ab,ti,kf.) AND (exp Neoplasms/ OR cancer*.ab,ti,kf. OR neoplas*.ab,ti,kf. OR carcinom*.ab,ti,kf. OR oncolog*.ab,ti,kf. OR malignan*.ab,ti,kf. OR tumor*.ab,ti,kf. OR tumour*.ab,ti,kf.))** | 538,753 |

**Embase.com Session Results (01 Dec 2022)**

| Search | Query | Items found |
| --- | --- | --- |
| #4 | **#1 AND #2 AND #3** | 5,552 |
| #3 | **'preoperative period'/exp OR 'neoadjuvant therapy'/exp OR 'preoperative':ab,ti,kw OR 'pre-operative':ab,ti,kw OR 'before surg*':ab,ti,kw OR 'neoadjuvant*':ab,ti,kw OR 'neo-adjuvant*':ab,ti,kw** | 792,201 |
| #2 | **'partial body radiation'/exp OR (('target*' OR 'partial*' OR 'segmental') NEAR/3 ('radiotherap*' OR 'radiati*' OR 'irradiati*' OR 'x ray therapy' OR 'x ray therapies')):ab,ti,kw** | 340,834 |
| #1 | **'breast tumor'/exp OR (('breast'/exp OR 'breast*':ab,ti,kw OR 'mamma*':ab,ti,kw) AND ('neoplasm'/exp OR 'cancer*':ab,ti,kw OR 'neoplas*':ab,ti,kw OR 'carcinom*':ab,ti,kw OR 'oncolog*':ab,ti,kw OR 'malignan*':ab,ti,kw OR 'tumor*':ab,ti,kw OR 'tumour*':ab,ti,kw))** | 819,152 |

**Web of Science (Core Collection) Session Results (01 Dec 2022)**

| Search | Query | Items found |
| --- | --- | --- |
| #4 | **#1 AND #2 AND #3** | 178 |
| #3 | **TS=("preoperative" OR "pre-operative" OR "before surg*" OR "neoadjuvant*" OR "neo-adjuvant*")** | 382,255 |
| #2 | **TS=((("target*" OR "partial*" OR "segmental") NEAR/3 ("radiotherap*" OR "radiati*" OR "irradiati*" OR "x ray therapy" OR "x ray therapies")) OR "partial body")** | 20,117 |
| #1 | **TS=(("breast*" OR "mamma*") AND ("cancer*" OR "neoplas*" OR "carcinom*" OR "oncolog*" OR "malignan*" OR "tumor*" OR "tumour*"))** | 760,903 |

**Scopus Session Results (01 Dec 2022)**

| Search | Query | Items found |
| --- | --- | --- |
| #4 | **#1 AND #2 AND #3** | 242 |
| #3 | **TITLE-ABS-KEY("preoperative" OR "pre-operative" OR "before surg*" OR "neoadjuvant*" OR "neo-adjuvant*")** | 574,495 |
| #2 | **TITLE-ABS-KEY((("target*" OR "partial*" OR "segmental") W/3 ("radiotherap*" OR "radiati*" OR "irradiati*" OR "x ray therapy" OR "x ray therapies")) OR "partial body")** | 25,593 |
| #1 | **TITLE-ABS-KEY(("breast*" OR "mamma*") AND ("cancer*" OR "neoplas*" OR "carcinom*" OR "oncolog*" OR "malignan*" OR "tumor*" OR "tumour*"))** | 776,824 |
